# Caspase dependent apoptosis is required for anterior regeneration in Aeolosoma viride and its related gene expressions are regulated by the Wnt signaling pathway

**DOI:** 10.1038/s41598-020-64008-1

**Published:** 2020-07-01

**Authors:** Sheridan Ke-Wing Fok, Chiao-Ping Chen, Tzu-Lun Tseng, Yi-Hua Chiang, Jiun-Hong Chen

**Affiliations:** National Taiwan University, Department of Life Science, Taipei city, 10672 Taiwan

**Keywords:** Developmental biology, Apoptosis, Regeneration

## Abstract

Although apoptosis has been widely observed during the regenerative process, the mechanisms by which it is regulated and its roles in regeneration remained unclear. In this study, we introduced *Aeolosoma viride*, a fresh water annelid with an extraordinary regenerative ability as our model organism to study the functions and regulations of apoptotic caspases. Here we showed that major events of apoptosis were detected near the wounded area and showed spatial correlation with the expression patterns of caspase gene namely *Avi-caspase X* and two apoptosis regulators namely *Avi-Bax* and *Avi-Bcl-xL*. Next, we investigated how *Avi-caspase X* gene expression and apoptosis influence regeneration following head amputation. RNA interference of *Avi-caspase X* reduced the amounts of apoptotic cells, as well as the percentage of successful regeneration, suggesting a critical role for apoptosis in anterior regeneration of *A. viride*. In addition, we also discovered that the expression of apoptotic caspases was regulated by the canonical Wnt signaling pathway. Together, our study showed that caspase dependent apoptosis was critical to the anterior regeneration of *A. viride*, and could be regulated by the canonical Wnt signaling pathway.

## Introduction

*Aeolosoma viride* is a fresh water annelid that can regenerate both anteriorly and posteriorly within 5 days after amputation^1^. Amputation created a rough and uneven wounded area that smoothened around 12 hours post amputation (hpa). After 12 hpa, a hyaline cell masses started to develop at the regenerating area which characterized blastema formation. Mouth formation was initiated around 96 hpa, in which a circular structure reappeared at the ventral side of the peristomium. After 96 hpa, the anterior regenerating head bulged and became wider than posterior segments. Most worms are freely to move at 120 hpa, which was considered as a successful anterior regeneration^2^. Previous studies showed that cell proliferation is required for the formation of blastema in *A. viride*^2^. Similar to many animal species, undifferentiated cells from the blastema can proliferate and differentiate in an orderly manner to replace the missing body parts, tissues or organs in this annelid^3–5^. Since many multicellular organisms maintain an appropriate body size, cell proliferation must be coordinated with other biological processes. One of such mechanisms is apoptosis^6^.

Apoptosis is a form of programmed cell death that eliminates cells that are damaged or no longer required in a coordinated manner^7^. In many organisms, apoptosis is a normal and essential part of development. Studies have confirmed that apoptosis is necessary for the proper development of many organisms for it affect a wide range of biological processes^8–11^. Organs and tissues that are only useful during the embryonic stage are eliminated by apoptosis to allow further development. Coordinated apoptosis on a large group of cells provide a mean to shape interdigital tissue in many vertebrate animals^12^. Apoptosis serve critical functions during development as well as regeneration^13^. For example, during the wound healing process of model organisms such as *Xenopus* and planarian, specific cell populations such as inflammatory and immune cells must be eliminated prior to the progression of regenerative response^14^. Following the completion of wound healing, blastemal cells undergo pattern formation to replace the missing structures in which the apoptosis participated by inducing cellular reorganization and terminates cell differentiation at aberrant positions^15^. In addition, apoptosis could also be the source of cell proliferation^13,16,17^. Apoptosis-induced compensatory proliferation coordinates cell death and cell proliferation through the Jun N-terminal kinase and p53 in *Drosophila*^18^.

The apoptotic processes involve a specific group of cysteine aspartic proteases (also known as caspases)^19,20^. Caspase proteins have been classified into three major groups, including inflammatory, initiator and effector caspases^19,21–23^ which differs in structure and domain composition. All caspases contained two essential catalytic domains: the p20 subunit and the p10 subunit which are derived from the p45 precursor^21,24^. Inflammatory caspases, which include caspases 1, 4, 5, 11 and 12 contain the p20 subunit and a caspase activation and recruitment domain (CARD) domain^25^. Initiator caspases, also contain the universal p20 subunit, but are different in their possession of CARD domain or death effector domain^26^. Initiator caspase 2 and 9 contains a CARD domain, while initiator caspase 8 and 10 contains death effector domain. Effector caspases are different from the inflammatory and initiator caspase in that it only possesses the p20 subunit. Apoptotic caspases (initiator and effector capsases) are known to regulate apoptosis through intrinsic and extrinsic pathways. In mammalian, intrinsic pathway initiates when cell senses internal stresses, then activates the downstream BH3 only molecule. The BH3 only molecule inhibits one of the major anti-apoptotic protein Bcl-xL, and activates one of the major pro-apoptotic protein Bax. Bax protein induces the release of cytochrome c to guild the formation of apoptosome. Apoptosome then cleaves the initiator caspase which activates effector caspase. On the other hand, extrinsic pathway takes place when Death ligand binds to Death receptor. The binding stimulates the adaptor protein Fas-associated death domain (FADD) protein to recruit the Death inducing signaling complex (DISC). Initiator caspase from DISC will then cleaves the effector caspase. Activated effector caspases degrade cellular components, breaking it into smaller pieces called the apoptotic bodies that will eventually be removed by macrophage^7,27^. Although caspases dependent apoptosis occurs normally in the developmental process of annelid^28–30^, its function and involvement during regeneration remain unclear. Our studies aim to investigate the function and the regulatory mechanism of caspase during anterior regeneration in *A. viride*. The association between the canonical Wnt signaling pathway and apoptosis has become more evident in recent literatures, studies have shown that apoptosis served as an unexpected sources of Wnt3 positive cell in hydra and Wnt11 induced cardiomyocyte development through caspase mediate suppression^31–33^. The canonical Wnt signaling pathway is known to plays significant role during regenerative process^34,35^, and interacts with many other signaling pathway to regulate biological processes^36,37^. To further understand the development and the evolutionary aspect of this relationship, our study also aims to investigate the association between apoptosis and canonical Wnt signaling pathway in this regenerating annelid species.

In this study, we described the spatiotemporal pattern of apoptosis during the regenerative process of *A. viride*. Furthermore, we identified two apoptotic caspases, *Avi-caspase Y* and *Avi-caspase X* that exhibited an elevated gene expression during anterior regeneration. RNA interference of *Avi-caspase X* significantly reduced the percentage of successful regeneration and the amount of apoptotic cells, demonstrating the importance of apoptosis during the regeneration process. Next, we identified the canonical Wnt signaling pathway as a regulator of caspase gene expression. When the worms were treated with XAV939, an inhibitor of the Wnt pathway, the gene expression of *Avi-caspase X* decreased significantly. However, *Avi-caspase X *RNA interference has no significant effect on the gene expression of *Avi-wnt-4*. *Avi-wnt-4* was previously identified as a key regulator of canonical Wnt signaling pathway^38^. Together these results suggested that gene expression of *Avi-caspase X* is critical to anterior regeneration of *A. viride*, and is regulated by the Wnt/β-catenin signaling pathway.

## Results

### Dynamic change of apoptotic cells during anterior regeneration

**Figure 1:**
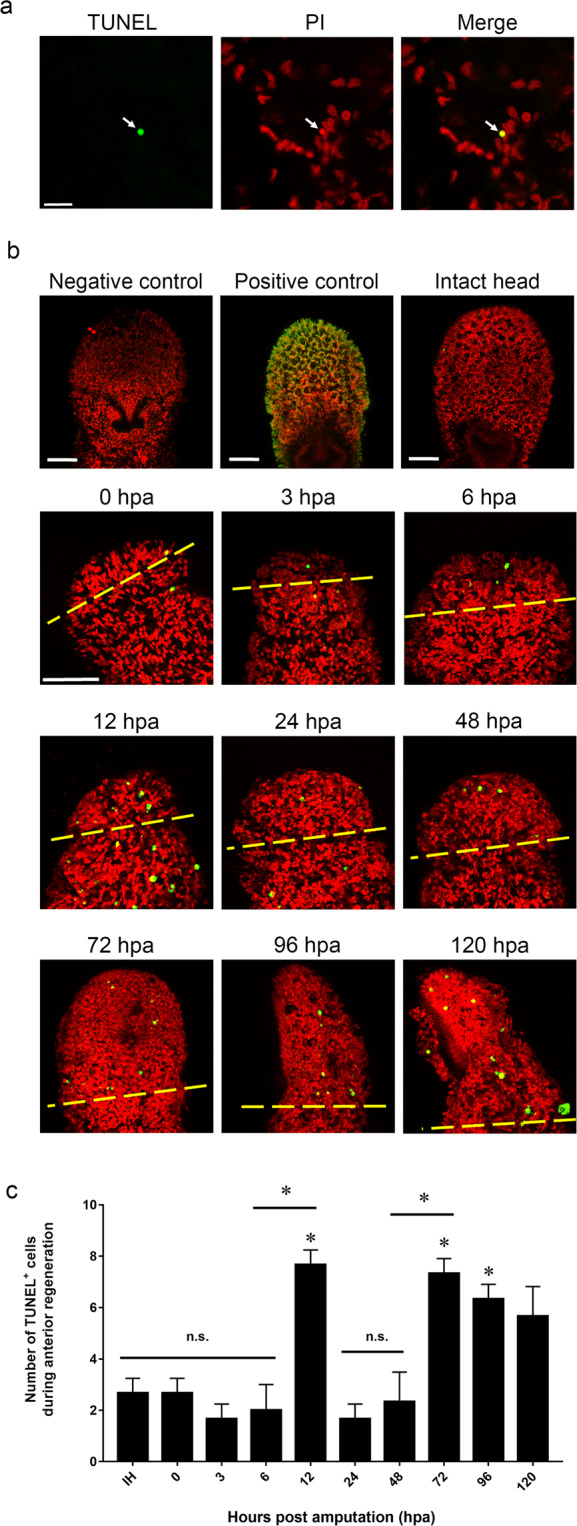
Detection of apoptotic cells during anterior regeneration. (**a**) TUNEL assays were performed to detect TUNEL^+^ cells (green), and cells were costained with PI (red). Co-localization of red and green signals, was easily recognized in yellow additive color (n = 5/group). (**b**) TUNEL^+^ cells detected at intact head and the anterior regenerating site. The yellow dotted line indicated the amputation site (n = 5/group). (**c**) Quantitative determination of TUNEL^+^ cells during each stage of anterior regeneration. All data represented the mean ± SD from three independent duplicate experiments. Significant differences relative to intact animals are denoted by *. *P ≤ 0.05 using the Mann Whitney U test. Scale bar: 50 μm.

In order to determine whether apoptosis takes place during the anterior regeneration of *A. viride*, Terminal deoxynucleotidyl transferase dUTP nick end labeling (TUNEL) assay was performed to label the apoptotic and necrotic cells at different stages of anterior regeneration. Apoptotic cells were recognized in yellow, as confirmed by the positive control, and the nuclei were counterstained in red by propidium iodide (PI), as confirmed by the negative control (Fig. S1). Cell positive for both TUNEL labeling and PI staining were considered as apoptotic cell (Fig. 1a). Our result indicated that apoptotic event was minimal at the intact head (IH) prior to amputation. Amputation was performed at the 4^th^ segment, and the number of apoptotic cells was recorded manually. No significant increase in the number of apoptotic cells was observed at the anterior regenerating site before 12 hpa. However, the number of apoptotic cells increased drastically at 12 hpa, and quickly diminished from 24 to 48 hpa. The number of apoptotic cells increased significantly again at 72 hpa, and remained elevated at 120 hpa (Fig. 1b). Two peaks of apoptosis were observed during anterior regeneration. The first peak occurred specifically at 12 hpa, a relatively early stage of regeneration. The second peak prolonged from 72 to 120 hpa. Since head formation initiate around 72 to 96 hpa and regeneration is completed around 120 hpa, stages after 72 hpa is considered to be the late stage of regeneration (Fig. 1c).

### Identification of Avi-caspase X, Avi-caspase Y, Avi-Bax, and Avi-Bcl-xL

**Figure 2:**
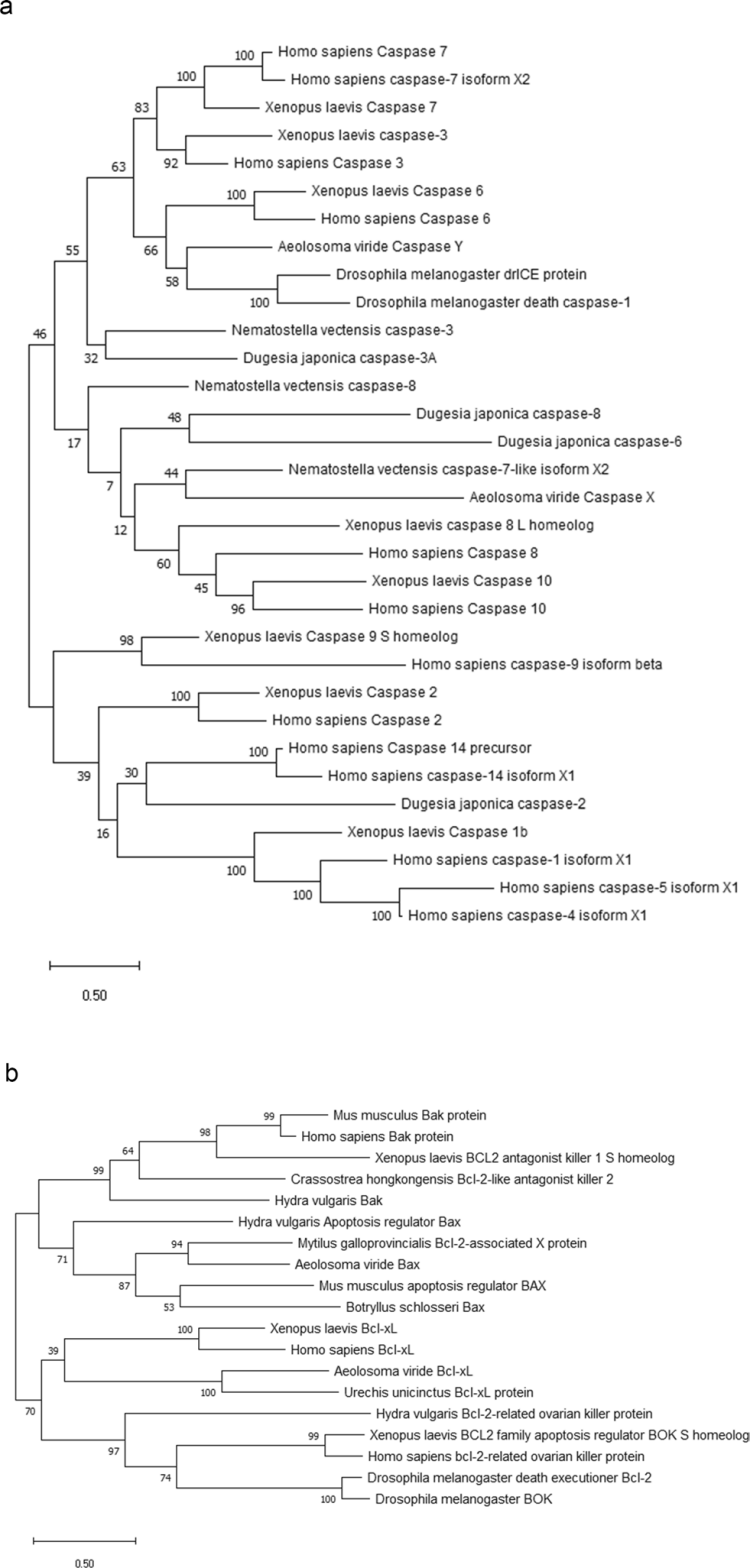
Phylogenetic tree for *Avi*-caspase X, Avi-caspase Y, *Avi*-Bax and *Avi*-Bcl-xL. (**a**) The phylogenetic tree for *Avi*-caspase X and *Avi*-caspase Y. This tree was constructed by amino acid sequences of the open reading frame (ORF). (**b**) The phylogenetic tree for *Avi*-Bax and *Avi*-Bcl-xL. This tree was constructed by amino acid sequences of the entire ORF.

An unpublished transcriptome database that composed of the regenerating tissue of three different stages (6, 24 and 48 hpa) and the intact head of the *A. viride* was coupled to rapid amplification of cDNA ends (RACE) that allowed us to obtain the complete sequence of two caspase genes and two Bcl-2 family genes. To further confirm the identities of these genes, phylogenetic trees were constructed using the conceptually translated *A. viride* proteins and other published protein sequences. In the tree of caspase proteins, effector caspases of *H. sapiens*, *X. laevis*, and *D. melanogaster* was grouped together. One of the caspase grouped with Caspase 6 of *H. sapiens*, *X. laevis*, *D. melanogaster*. A branch point separated the deuterostome and protostome which is largely consistent with the consensus phylogeny. Sequence analysis showed that that this caspase contains the p20 subunit, the CASc family domain, and a QACXG (where X is R, Q, or G) pentapeptide active site motif (Fig. S2b) that is found in most effector caspases. However, it did not form a monophyletic group with any known effector caspase sequence, thus was named *Avi-caspase Y*. The other caspase sequence of *A. viride* formed a monophyletic group with caspase-7-like isoform X2 of *Nematostella vectensis*, however it was not separated from the initiator caspases of *H. sapiens*, *X. laevis*, and *Dugesia japonica*. Phylogenetic analysis showed that this caspase share the highest sequence identity with apoptotic caspases, such as caspase −7 of *Strongylocentrotus purpuratus* (XP_011676882.1), caspase 3/9 of *Patiria pectinifera* (ACM46824.1), and caspase-3-like of *Acanthaster plancic* (XP_022090365.1). All four caspases are identified with a CASc domain, which is approximately 200 amino acids in length. In addition, two essential catalytic domains: the p20 subunit and the p10 were also identified in this novel caspase. This novel caspase sequence found in *A. viride *only contains the p20 subunit, suggesting it being an effector caspase (Fig. S2a). Based on the structure profile and the outcome of phylogenetic analysis, this effector caspase was thus named *Avi-caspase X* (Fig. 2a).

A different phylogenetic tree was also constructed for the two sequences of Bcl-2 family protein. The pro-apoptotic protein Bax and BAK divided into two cluster, separated from the anti-apoptotic protein Bcl-xL, Bcl-2, and BOK. Bax protein of *A. viride* group with the Bax protein cluster, and showed the highest sequence similarity to the mollusca *Mytilus galloprovincialis*. On the other hand, protein sequence of Bcl-xL separated into two clusters, Bcl-xL of vertebrate animals such as *Homo sapiens*, *X. laveis*, and *Mus musculus* were grouped together. Bcl-xL of invertebrate animals included two species of annelid: *A. viride* and *Urechis unicinctus* were grouped together (Fig. 2b).

### Gene expression of *Avi-caspase X*, *Avi-caspase Y*, *Avi-Bax*, and *Avi-Bcl-xL* during anterior regeneration

**Figure 3:**
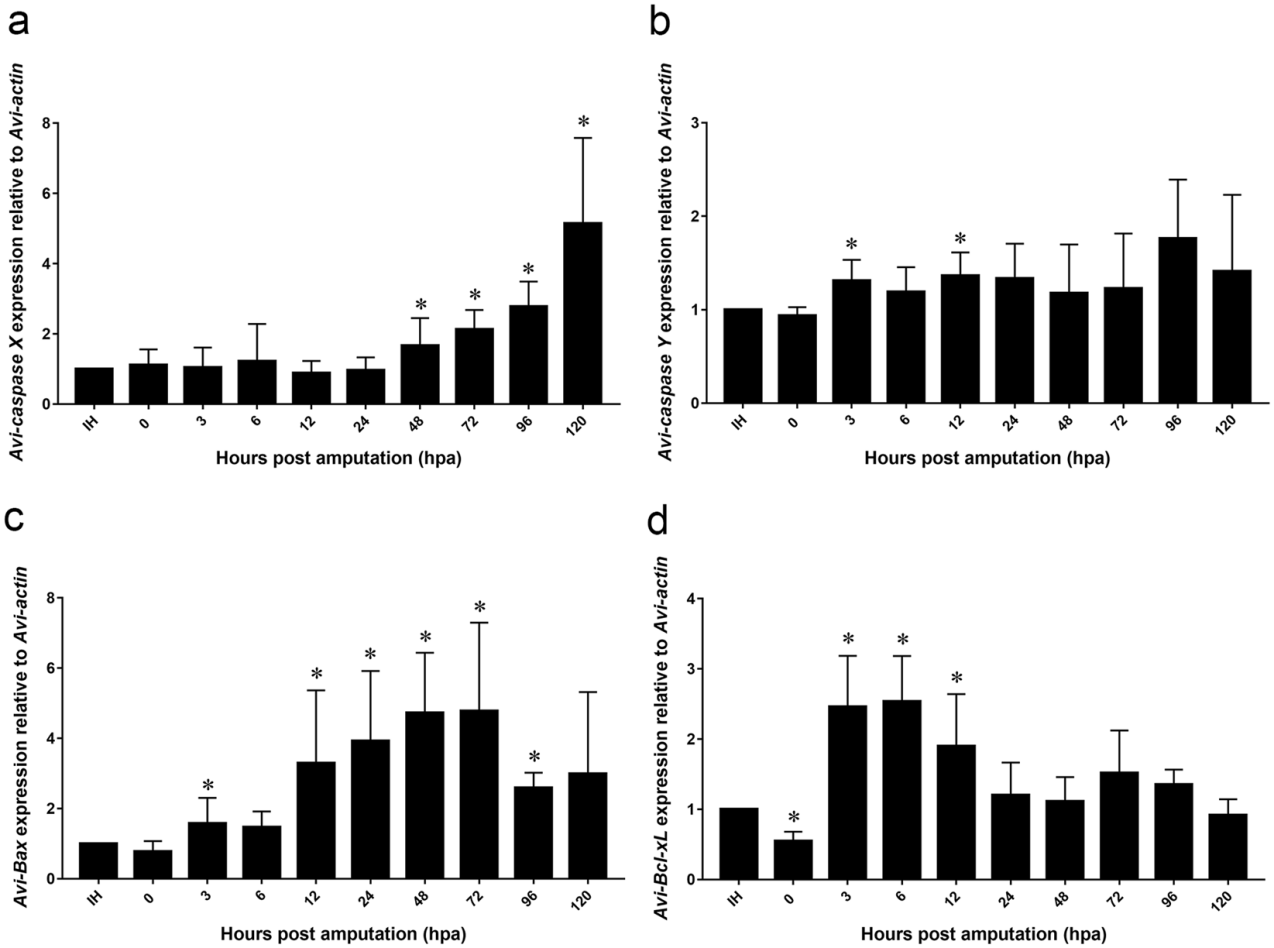
Relative gene expression of apoptotic related genes during anterior regeneration. mRNA gene expression of *Avi-caspase X* (**a**), *Avi-caspase Y* (**b**), *Avi-Bax* (**c**), *Avi-Bcl-xL*(**d**) during anterior regeneration in *A. viride*. The expression level was first normalized to *Avi-actin* and then to the normalized value of the intact head (IH). All data represented the mean ± SD from at least three independent duplicate experiments. Significant differences relative to intact are denoted by *. *P ≤ 0.05 using Mann Whitney U test.

To examine the involvement of these apoptosis related genes during anterior regeneration, qPCR was performed to measure the change in mRNA expression level. In order to minimize interfering signals from the intact body, regenerating tissues at the regeneration site was collected for detection. Regenerating tissues is not visible prior to 6 hpa, therefore two anteriormost segments at the amputation site was collected. Relative to the intact head, the gene expression of *Avi-caspase X* in the blastema had no significant difference before 48 hpa, but the gene expression significantly increased after 72 hpa, and reached its maximum around 96 to 120 hpa (Fig. 3a). A distinct expression pattern of *Avi-caspase Y* in the regenerating tissues was detected during anterior regeneration. The gene expression of *Avi-caspase Y* elevated during 3 and 12 hpa. The gene expression declined to its initial level around 48 to 72 hpa, and appeared to display an increasing trend at 96 hpa (Fig. 3b). Gene expression of pro-apoptotic gene *Avi-Bax *in the regenerating tissues showed no significant difference before 6 hpa. But the gene expression significantly increased after 12 hpa, and reached its maximum around 48 to 72 hpa. The gene expression decreased significantly after 96 hpa, and gradually returned to baseline (Fig. 3c). The anti-apoptotic gene, *Avi-Bcl-xL* showed a completely different expression pattern from *Avi-caspase X*, *Avi-caspase Y* and *Avi-Bax*. Gene expression of *Avi-Bcl-xL *slightly declined immediately after amputation, but significantly increased after 3 hpa, and reached its maximum around 3 to 12 hpa. After 12 hpa, the gene expression decreased significantly, and showed no significant difference with the IH from 48 hpa to 120 hpa (Fig. 3d).

### Location of gene expression of *Avi-caspase X* and *Avi-caspase Y* at the regenerating tissuesduring anterior regeneration

**Figure 4:**
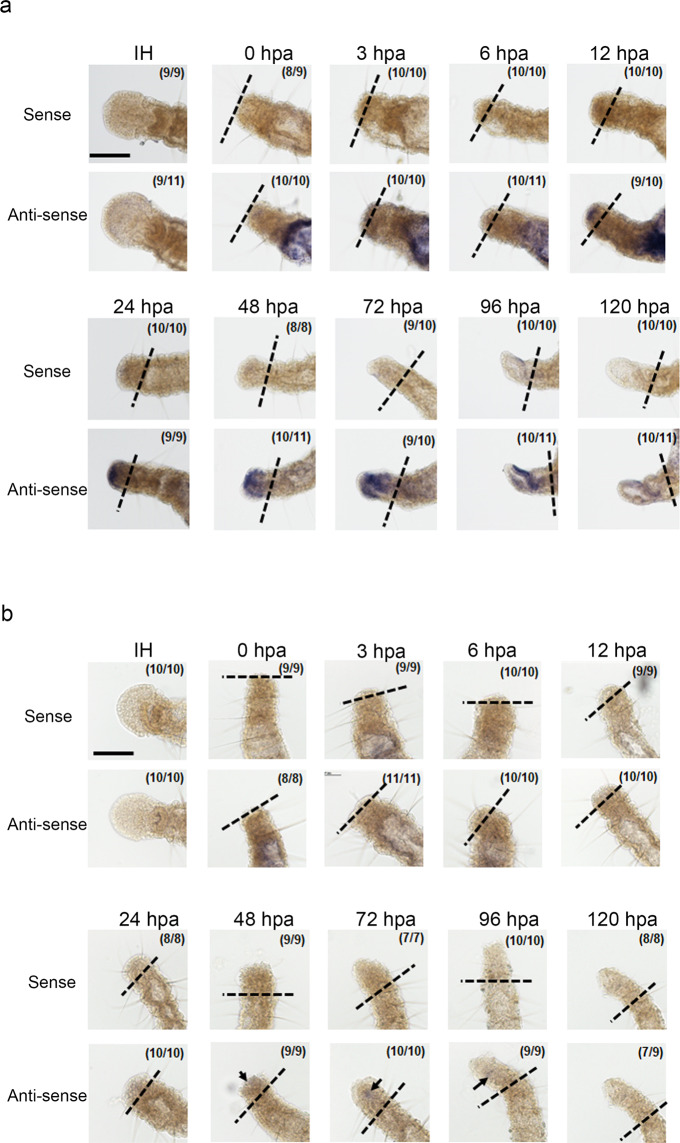
Localization of *Avi-caspases* during anterior regeneration. *Avi-caspase X* (**a**) and *Avi-caspase Y* (**b**) *in situ* hybridizations performed on intact and regenerating animals, with sense probe used as negative control. Black dotted line indicated the amputation site. Minimal signals could be detected around 48–96 hpa (arrow head). Scale bar: 100 µm.

To identify the location of *Avi-caspase X* expression in the anterior regeneration of *A. viride*, whole-mount *in situ* hybridization (WMISH) was performed on *A. viride *at different stages of regeneration. No specific signal was detected in the anterior region of intact worms. However, consistent expression was detected at the gut, which might be indicative of the rapid turnover of intestine lining. Starting from 12 hpa, the staining signal became visible at the tip of anterior regenerating site. This signal persisted and reached its relative maximum around 72 hpa. A specific expression pattern was observed at 96 hpa; the signal was expressed specifically on one side of the regenerating head, and extended into a tubular structure, which appeared to resemble the opening of the digestive tract. The signal gradually decreased after 120 hpa (Fig. 4a).

WMISH was also performed to detect the expression of *Avi-caspase Y*. Similar to *Avi-caspase X*, no specific signals were detected at the anterior region of intact worms. *Avi-caspase Y *expression was detected at the gut region prior to 24 hpa. Extremely weak signals became visible at the tip of anterior regeneration site around 48 to 72 hpa. Signals could no longer be detected at the anterior regenerating site after 96 hpa (Fig. 4b). Both qPCR and WMISH experiments showed that *Avi-caspase X* expression is highly correlated to the process of anterior regeneration, therefore we focused on *Avi-caspase X* in subsequent experiments.

### Gene expression of *Avi-caspase X* is critical to apoptosis

**Figure 5:**
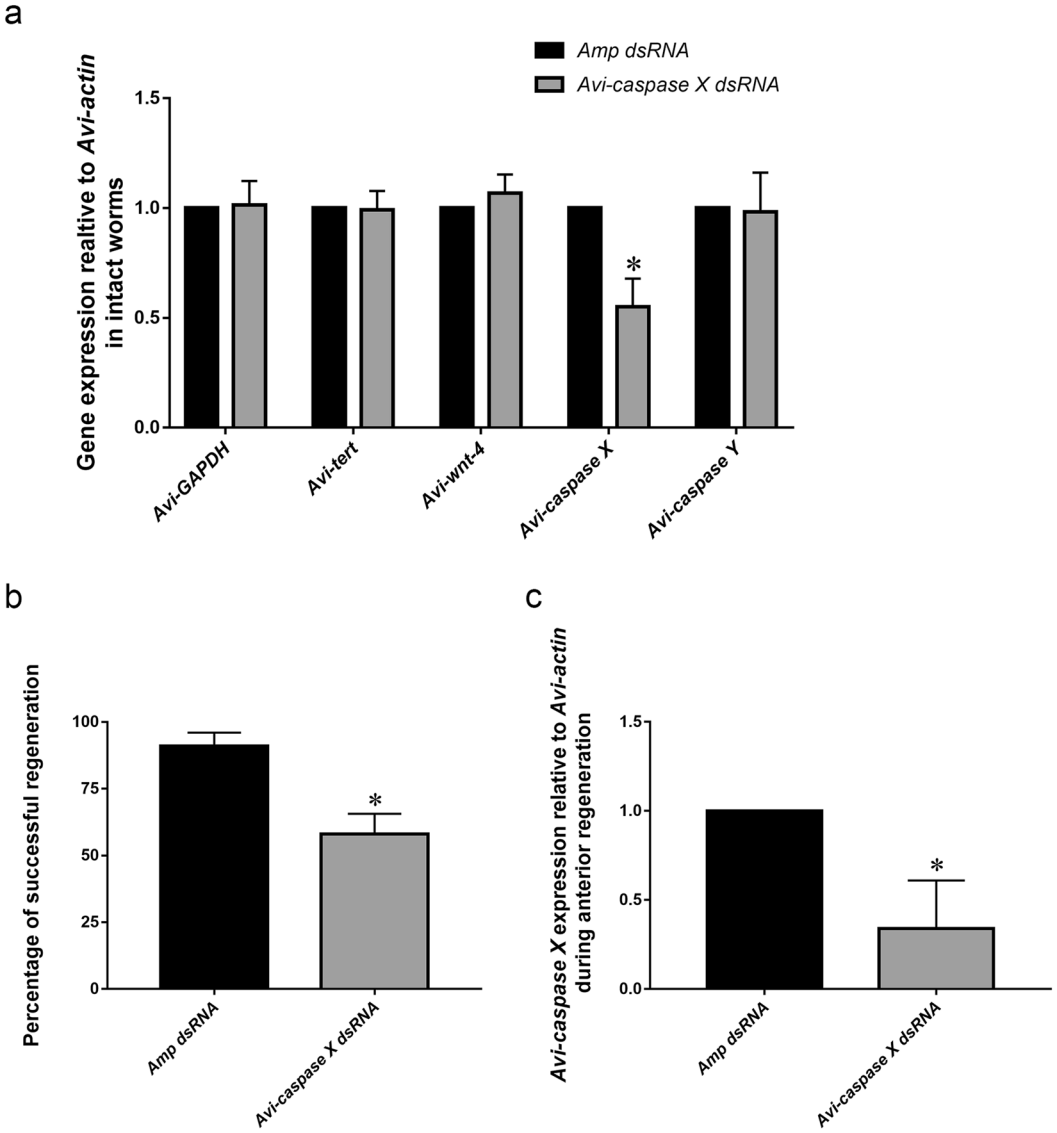
Knock-down experiment of *Avi-caspase X* by dsRNA. (**a**) mRNA gene expression of *Avi-GAPDH*, *Avi-tert*, *Avi-caspase X, Avi-caspase* and *Avi-wnt-4* was measured after dsRNA microinjection. (**b**) The percentage of successful regeneration was measured at 120 hpa. (**c**) mRNA gene expression of *Avi-caspase X* was measured after 120 hpa. All data represented the mean ± SD from at least three independent duplicate experiments. *P ≤ 0.05 using Mann Whitney U test.

**Figure 6:**
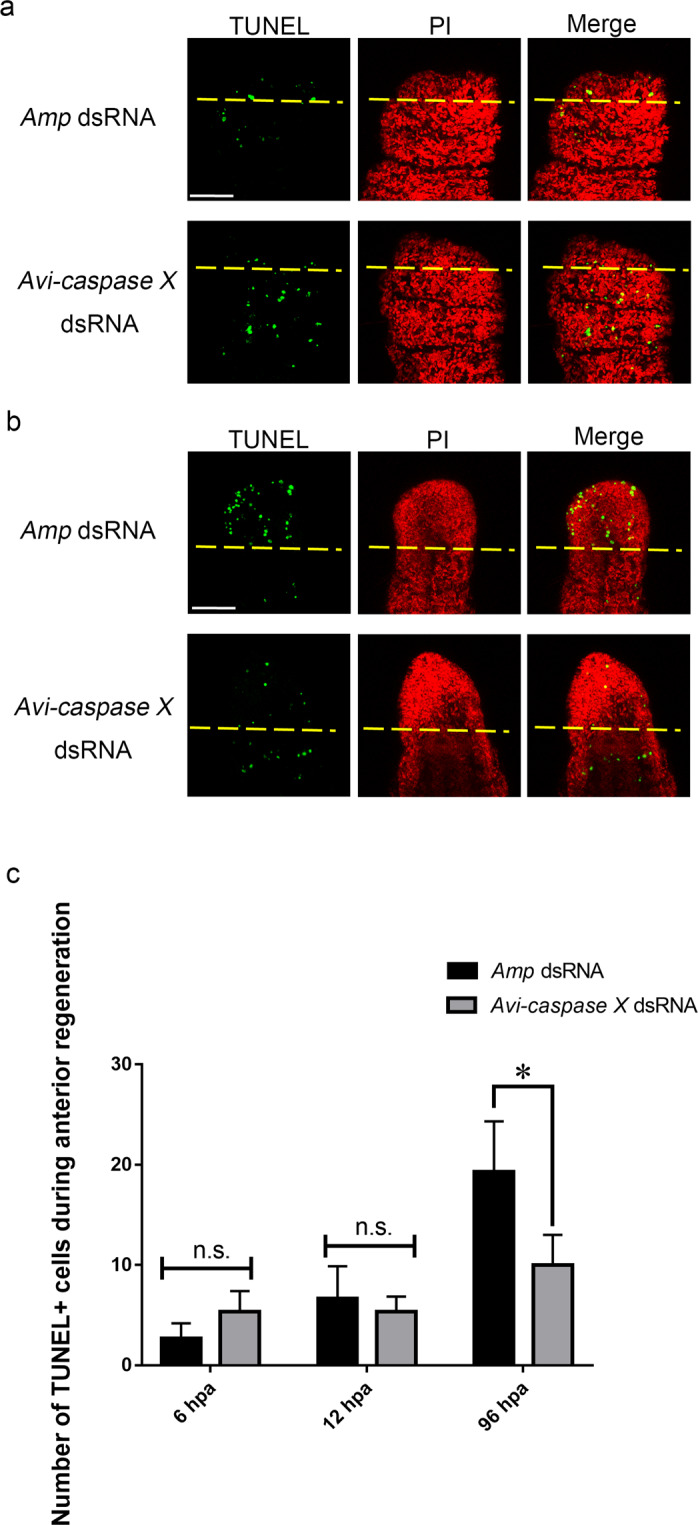
Apoptotic signals decreased after dsRNA treatment. TUNEL assays were performed to detect apoptotic signal at 12 hpa (**a**) and 96 hpa (**b**) after treated with dsRNA. (**c**) Quantitative determination of TUNEL^+^ cells during each stage of anterior regeneration (n = 5/group). All data represented the mean ± SD from three independent duplicate experiments. Significant differences are denoted by *.*P ≤ 0.05 using Mann Whitney U test. Scale bar: 50 μm.

In order to determine whether *Avi-caspase X* is required for anterior regeneration, RNAi was performed to knockdown gene expression. To successfully deliver RNAi, a microinjection method was developed for this model organism. To validate this injection method, we injected ASW and monitored its effect on the regenerating *A. viride*. Our experiment showed no significant difference in the regenerating process of animals injected with ASW or no fluid. And to tract the delivered fluid, we injected the worms with a 200 times diluted cherry red in ASW. The slightly magenta fluid was overserved to circulate the worms’ body cavity (data not shown). For the RNAi experiment, the bacterial a*mpicillin resistant gene* (amp)was used as control RNAi. Relative gene expression and the percentage of successful regenerationwas recorded after the RNAi treatment was applied. Animals were injected with 100 ng of double stranded RNA (dsRNA) for 2 consecutive days. Gene expression of *Avi-caspase X* significantly decreased after the injection in intact animals (Fig. 5a). The mRNA expression of housekeeping gene *Avi-GAPDH* and the telomerase gene *Avi-tert* showed no significant change after *Avi-caspase X *dsRNA injection, which confirmed the specificity of our dsRNA. *Avi-caspase X *dsRNA also showed no significant effects on the gene expression of *Avi-caspase Y* and *Avi-wnt-4*. (Fig. 5a). To test if *Avi-caspase X* is required for anterior regeneration, worms were amputated after two consecutive days of dsRNA injection. Worms injected with *Avi-caspase X* dsRNA showed significant lower percentage of successful regeneration at 120 hpa (Fig. 5b). Given the high variability in the dynamics of dsRNA up-taking in the worms, gene expression was measured at a single time point (120 hpa). It was found that worms did not regenerate well also showed significantly lower levels of *Avi-caspase X *expression (Fig. 5c). To further validate the requirement of *Avi-caspase X* gene in apoptotic activities during regeneration, TUNEL assay was performed after dsRNA injection. The amount of apoptotic cells decreased to approximately 50% of the control group in the group treated with *Avi-caspase X *dsRNA at 96 hpa (Fig. 6).

Together, our data suggested that apoptosis was involved in the process of regeneration. In addition, the gene expression of *Avi-caspase X* is required for caspase dependent apoptosis and proper regeneration in *A. viride*.

### Wnt signaling pathwayis required for anterior regeneration in *A. viride*

**Figure 7:**
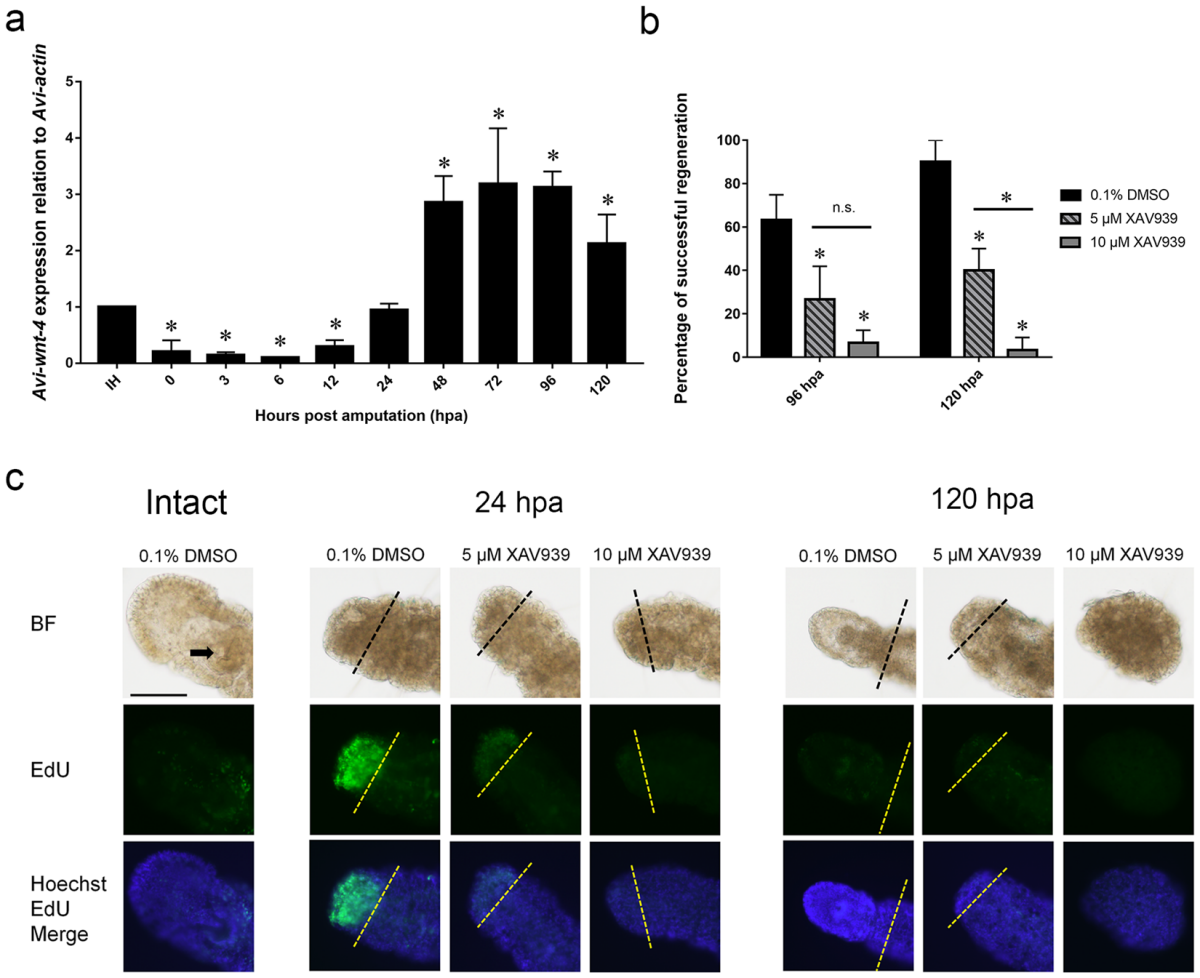
*Avi-wnt-4* is required during anterior regeneration in *A. viride*. (**a**) mRNA gene expression of *Avi-wnt-4* during anterior regeneration in *A. viride*. All data represented the mean ± SD from at least three independent duplicate experiments. (**b**) Dose-dependent experiment of XAV939. 0.1% DMSO in ASW was used as control group (n = 10/group). (**c**) EdU^+^ detection in regenerating *A. viride* incubated in 0.1% DMSO in ASW or treated with either 5 μM or 10 μM of XAV939 for 5 consecutive days after amputation (n = 10/group). Worms were exposed to 0.1 mM of EdU in ASW for 12 hours. All data represented the mean ± SD from three independent duplicate experiments. Significant differences relative to 0.1% DMSO were denoted by *; Significant difference between treatment groups was specified by black solid line and denoted by * for (**b**). *P ≤ 0.05 using Mann Whitney U test. Scale bar: 50 μm.

Previous studies showed that *Avi-wnt-4* is required for anterior regeneration in *A. viride*^38^. To confirm that *Avi-wnt-4* participates in anterior regeneration of *A. viride*, qPCR was performed to detect the change in *Avi-wnt-4* mRNA expression level during the course of regeneration. The expression levels of *Avi-wnt-4* in the blastema decreased immediately after amputation. After 24 hpa, however, the levels of gene expression gradually increased and became statistically significant after 48 hpa. The expression level of *Avi-wnt-4* reached its maximum at around 72 hpa, and then gradually decreased (Fig. 7a). An inhibitor treatment experiment was next performed to test the role of Wnt signaling pathway during anterior regeneration. XAV939, a Wnt signaling pathway inhibitor which stimulates β-catenin degradation by stabilizing Axin, was applied to the worms at different concentrations. Our results showed that XAV939 has asignificant inhibitory effect on regeneration in a dosage-dependent manner. The percentage of successful regeneration at 120 hpa decreased from 90% in the control to 40% in the group treated with 5 μM of XAV939. The effect was even more pronounced in the treatment group applied with 10 μM of XAV939, where its percentage of successful regeneration decreased to less than 5% (Fig. 7b). Finally, EdU labeling was used to characterize how XAV939 inhibits regeneration. At 24 hpa, there were no specific morphological differences between the control and the group treated with different concentration of XAV939. However, there was significant difference in the amount of EdU^+^ cells between groups. In worms treated with 5 or 10 μM of XAV939, there was no EdU signals at the anterior regenerating site, indicating that the blastema formation was inhibited. At 120 hpa, regeneration had proceeded normally in worms treated with 0.1% DMSO, as a bulged head and mouth was observed. In contrast, no head had been regenerated in groups treated with 5 μM of XAV939. In groups treated with 10 μM of XAV939, the body of the worm degenerated into a tiny piece of tissue, in which the head and the posterior structures could no longer be identified, and the segmental organization and the setae of the trunk were largely lost (Fig. 7c).

### The effect of XAV939 on apoptosis during regeneration

**Figure 8:**
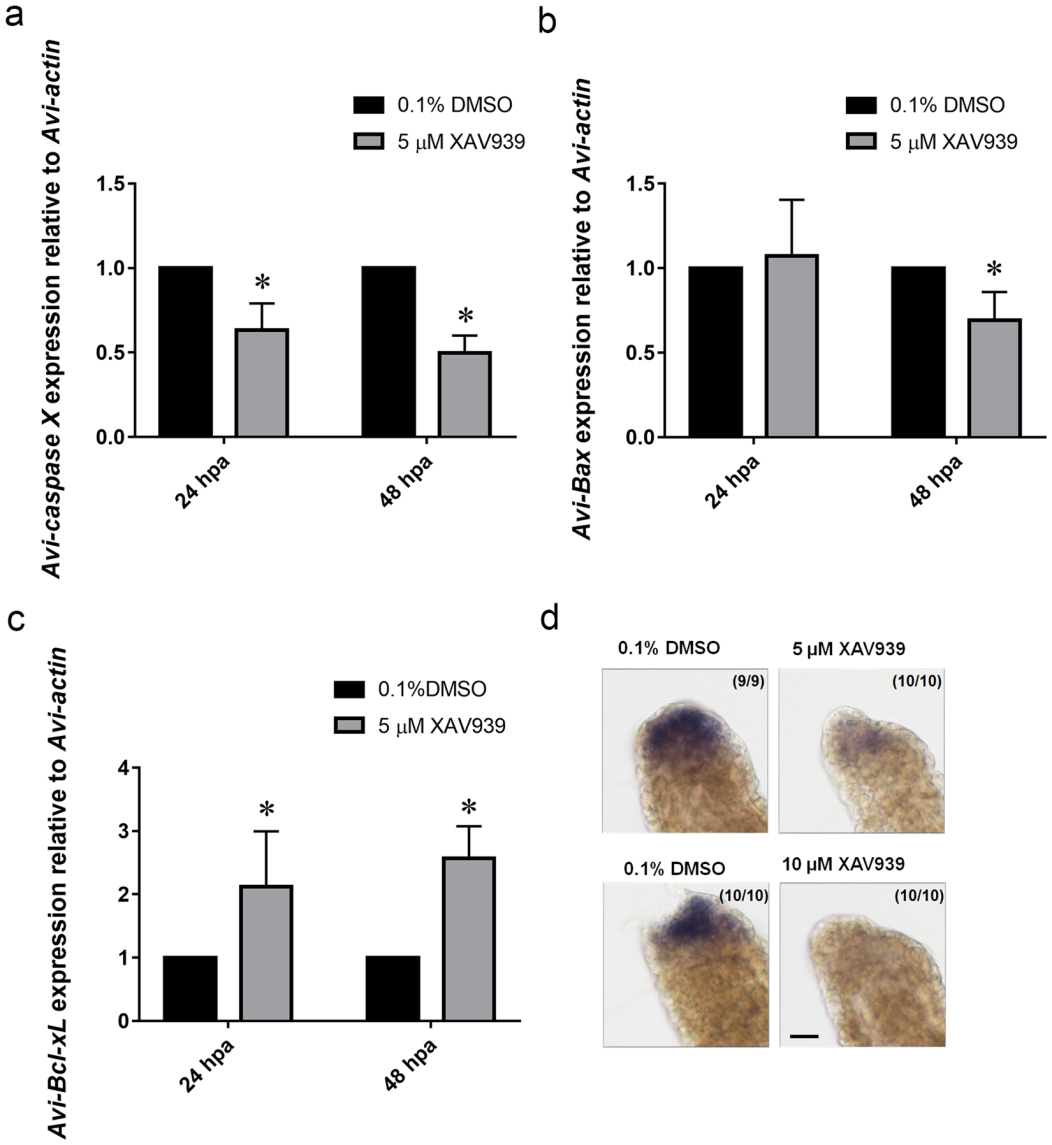
Gene expression of *Avi-caspases*, *Avi-Bax*, *and Avi-Bcl-xL* decreased under XAV939 treatment. mRNA gene expression of *Avi-caspase X* (**a**), *Avi-Bax *(**b**), and *Avi-Bcl-xL *(**c**) after treated with XAV939 during critical period. (**d**) *In situ* hybridizations were performed afterward to detect *Avi-caspase X* expression at 48 hpa. All data represented the mean ± SD from at least three independent duplicate experiments. Significant differences relative to 0.1% DMSO are denoted by *. *P ≤ 0.05 using Mann Whitney U test. Scale bar: 50 µm.

**Figure 9:**
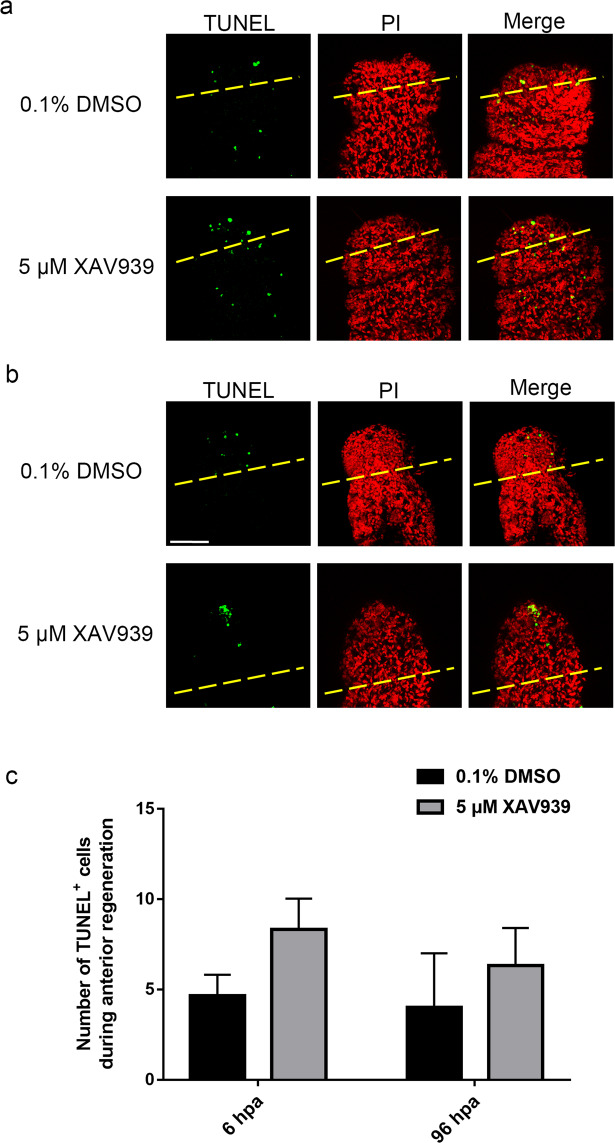
Apoptotic signals increased after XAV939 treatment. TUNEL assays were performed to detect apoptotic cells (green), and cells were costained with PI (red). Colocalization of red and green signals, was easily recognized in yellowadditive color. TUNEL assays were performed to detect apoptotic signal at 6 hpa (**a**) and 96 hpa (**b**) after treated with 5 µM of XAV939 in 0.1% DMSO (n = 5/group). Yellow dotted line indicated the amputation site. Quantitative determination of TUNEL^+^ cells during 6 and 96 hpa after treated with XAV939 (n = 5/group). All data represented the mean ± SD from three independent duplicate experiments. Significant differences are denoted by *. *P ≤ 0.05 using Mann Whitney U test. Scale bar: 50 μm.

To determine if the Wnt signaling pathway regulates the expression of apoptotic caspases, the expression levels of *Avi-caspase X* was probed after XAV939 treatment. Both qPCR and *in situ* hybridization experiments revealed a significant decrease in *Avi-caspase X* gene expression after being treated with 5 μM of XAV939 (Fig. 8a,d). To see if other components of the apoptotic pathway are regulated by the Wnt signaling pathway interacts with the apoptotic caspases, the expression levels of the pro-apoptotic *Avi-Bax *and the anti-apoptotic *Avi-Bcl-xL *following XAV939 were revealed by qRT-PCR. When the worms were treated with 5 μM of XAV939, *Avi-Bax *was down-regulated significantly while *Avi-Bcl-xL *was up-regulated (Fig. 8b,c). These findings implied that Wnt signaling pathway can promote apoptotic activity during regenerationby regulating the expression of genes in the apoptotic pathway. Therefore, we went on to see if XAV939 treatment inhibits apoptotic activity by TUNEL assay. However, unexpectedly, there was no decrease in the number of apoptotic cells in worms treated with XAV939. There was no significant difference in the number of apoptotic cells between worms treated with control or 5 μM of XAV939, only a tendency of increase could be observed after the XAV939 treatment (Fig. 9a–c). Together, these data confirmed that the canonical Wnt signaling pathway is required for proper regeneration in *A. viride*. And the Wnt signaling pathway can regulate the gene expression of *Avi-caspase X* and *Avi-caspase Y* by down-regulating the gene expression of *Avi-Bax* and up regulating the gene expression of *Avi-Bcl-xL*.

## Discussion

Apoptosis is critical to the proper development and regenerative process in many model organisms. It serves as a control mechanism to remove dysfunctioning, unwanted or damaged cells from the tissues or organs^13,39,40^. Recent findings have shown that apoptosis can even be the driving force of cell proliferation during tissue regeneration^16,18,41,42^. In this study, apoptosis was found to be involved in regeneration of *A. viride*. Two distinct waves of apoptotic reactions were observed during the process of anterior regeneration in *A. viride* at 12 hpa and 72 to 120 hpa. This is similar to what had been observed in the regenerating fragments of planarian, in which an initial apoptotic response peaked at approximately 1 to 4 hours after injury, and is followed by a second systemic response that peaked 3 days after amputation^43^. The two rounds of apoptosis during regeneration was also observed in Porifera *Aplysina cavernicola *and the caudal regeneration of zebrafish^41,44,45^.

Generally, *g*ene expression of *caspase* is elevated during regeneration in many species^46–48^. We showed that, similarly, *Avi-caspase X* expression is elevated in the anterior regenerating site of *A. viride*. During the process of anterior regeneration, the expression level of *Avi-caspase X* increased significantly from 48 hpa on and remained elevated until 120 hpa. Since *A. viride* could complete anterior regeneration five days post amputation (dpa) and head formation initiates around 72 hpa, 72 hpa and 96 hpa are considered to be the late stage of regeneration. This “late stage” expression was also observed in planarian and hydrae^36,49,50^. During late stage of regeneration, wound healing and blastemal formation are mostly completed while differentiation and pattern formation take over in succession^51^. Because apoptosis is also known to be involved in the process of pattern formation during organogenesis^52^, it is plausible that the late-stage expression of *Avi-caspase X* is involved in pattern formation during anterior regeneration. It is known that the upregulation of caspase gene is crucial to proper regeneration in other animal species^31,53,54^. When the worms were treated with *Avi-caspase X *dsRNA, the percentage of successful regeneration significantly decreased, and the amounts of apoptotic cells were significantly reduced in the late stage of regeneration (96 hpa). Together, it is reasonable to infer that *Avi-caspase X* is critical to the late stage apoptosis and regeneration in *A. viride*.

*Avi-caspase X* and *Avi-caspase Y* were both effector caspases but appeared to have differing functions during the regenerative process. Based on the *in situ* hybridization results, *Avi-caspase Y* only expressed at a minimal level at the blastema region during anterior regeneration. Similar results were obtained by using qPCR, since the expression levels of *Avi-caspase Y* have never increased more than 1.5 folds above that of the intact head. Therefore, the dynamics of gene expression was quite different between *Avi-caspase Y* and *Avi-caspase X*. These data suggested that *Avi-caspase X* is apparently more involved in the process of anterior regeneration than *Avi-caspase Y* is.

The elevated gene expression and the increase in number of apoptotic cells evident the involvement of caspase dependent apoptosis in the anterior regeneration of *A. viride*. This was further supported by the qPCR results on *Avi-Bax* and *Avi-Bcl-xL*. Bax and Bcl-XL are both bcl-2 family proteins, and serve critical function in caspase dependent apoptosis. In our experiment, the increased apoptosis was always accompanied by the up-regulation of both caspases genes and an increased expression of the pro-apoptotic gene *Avi-Bax*. On the other hand, the decreased or inhibited apoptosis was accompanied by the down-regulation of *Avi-caspase X* and an increased expression of the anti-apoptotic gene *Avi-Bcl-xL*. This finding are comparable to many other model organisms^55,56^.

Recent studies revealed a possible regulatory relationship between the canonical Wnt signaling pathway and apoptosis^32,57,58^. Apoptosis was found to be necessary to induce Wnt3 production and head regeneration in hydrae^31^, which indicates that Wnt3 expression might induced by apoptotic activity. On the other hand, the Wnt/β-catenin signaling promotes apoptosis in RAW264.7 macrophages^58^, suggesting that caspase might be the downstream target of Wnt signaling pathway. In this study, we found that the Wnt inhibitor has a significant inhibitory effect on both the expression levels of *Avi-caspases *and the rate of successful regeneration. The expression levels of apoptosis related genes *Avi-Bcl-xL* and *Avi-Bax* were also measured. As expected, a down-regulation of pro-apoptotic gene along with an up-regulation in anti-apoptotic gene was observed. On the other hand, the expression levels of *Avi-wnt-4* had not changed significantly after *Avi-caspase X* knockdown, indicating that *Avi-caspase X* could be the downstream targets of the Wnt signaling pathway in *A. viride*. But further experiment on other Wnt genes must be conducted to confirm this relationship.

Interestingly, the number of apoptotic cells did not significantly change after treated with XAV939, although expression of caspases and pro-apoptosis genes was inhibited by XAV939 (Figs. 8 and 9). This finding suggested that apoptosis could be induced partially independent of a high level of *Avi-caspase X* expression. Since lophotrochozoans typically contains a large number of caspasegene, we are anticipating the possibility of other un-discovered caspase sequence in this model organism^59,60^. Although the number of apoptotic cells did not significantly change after XAV939 treatment, a tendency of increase in apoptotic cells was observed (Fig. 9a–c). This surprise increase in number of apoptotic cells after XAV939 treatment might be contributed by other caspase genes as well. In addition, caspase-independent apoptosis which requires apoptosis inducing factor (AIF-1) but not caspase had been discovered for more than two decades^61,62^, and a homolog of AIF-1, a component of the caspase-independent pathway, was identified in the transcriptome of *A. viride*. It will be interesting to see if caspase-independent apoptosis and AIF-1 are involved in the regenerative process of *A. viride*.

Apoptosis, a form of programmed cell death that is conserved across evolution, is critical for the removal of unwanted or damage cells^63^. However, apoptosis has demonstrated different patterns and involvements in the regenerative process of model organisms. Planarian showed two waves of apoptosis during regeneration, and was believed to have possible roles in tissue remodeling^39^. Similarly, two rounds of apoptosis have been described in the caudal fin regeneration of zebrafish^41^, and anterior regeneration of *A. viride*, a pattern that has not yet been described in the study of model organisms such as Hydra and *Drosophila*. In addition, caspase-like genes are activated, and apoptotic cell are present throughout the regenerative process in planarian^50^, which is similar to *A. viride* that showed caspase correlated apoptosis at the amputation site. Amputation also triggers apoptosis in hydra, but was only overserved in the regenerating head. Foot regeneration in hydra appears to be independent of apoptosis and caspase expression^31^, which greatly differs from planarian and *A. viride*. The difference in involvements and patterns tempting us to speculate that apoptosis evolved to serve different roles in the regenerative process, but further investigation on the evolution of regeneration is required.

## Methods

### Animal cultures and regeneration procedure

*Aeolosoma viride* was cultured in artificial spring water (ASW, 48 mg/L NaHCO_3_, 24 mg/L CaSO_4_•2H_2_O, 30 mg/L MgSO_4_•7H_2_O, and 2 mg/L KCl in distilled water, pH = 7.4) at 25 °C under 12 hours of day-night cycles. Grounded oat meal was provided to the worms as primary food source. 20 mg of oat meal was fed to 500 ± 200 worms 3 to 5 times per week. In all regeneration experiment described here, only worms capable of producing zooid (older than 1 week) and of similar size (2.7 ± 0.2 mm) were selected for further experiment. Animal preparation and basic experimental procedures were as described in previous study^1^. Briefly, the experimental procedures began with starvation in ASW overnight prior to amputation. To minimize the influence of asexual reproduction, segments posterior to the midgut were amputated. After 3 days of recovery, anterior regeneration was initiated by amputating four anterior segments including prostomium, peristomium and three segments with chaetae. Regenerating worms were collected for the following 0 to 5 days after amputation. Specimens were examined live (in ASW) or fixed in 4% paraformaldehyde (PFA) using dissecting microscope (WILD M8, Leica) for detail morphological observation. Fixation method were as described in our previous studies^1,2^.

### Drug treatment

XAV939 (Sigma-Aldrich) was dissolved in dimethyl sulfoxide (DMSO) as stock solutions, and then diluted with ASW as working solutions in desired concentrations. Amputated worms were transferred into fresh ASW or ASW containing 10 or 5 μM XAV939 in 0.1% DMSO for subsequent experiments. The compartment was kept at dark, and the drug was replaced each day to maintain its effectiveness.

### TUNEL assay

Terminal deoxynucleotidyl transferase (TdT) dUTP nick-end labeling (TUNEL) assay were performed using ApopTag Plus Fluroescein *In situ* Apoptosis Detection Kit (Merck Millipore). Samples were collected 24 hours prior to the experiment and fixed in 4% PFA at 4 °C. Samples were washed with phosphate buffered saline with 0.1% Tween-20 (PBS-T, pH 7.4) for 5 times. After wash, sample were treated with 10 mg/ml proteinase K in PBS-T buffer for 10 minutes. The proteinase K digested samples were re-fixed in 4% PFA for 20 minutes on shaker, then washed with PBS-T 5 times to remove residual PFA. As positive control, worms were pre-incubated with 2.5 U DNase I (Promega) for 30 minutes at 37 °C. Subsequently, treated sample were washed with PBS-T for 2 times prior to the addition of reaction buffer containing nucleotide mix and TdT enzyme. Samples were incubated in the incubation buffer for 2 hours at 37 °C. After incubation, anti-DIG conjugate was added to the sample and incubated for 12 hours in dark at 4 °C. After the removal of anti-DIG conjugate, the sample was washed off with PBS-T for 5 time. PBS-T containing 0.5–1.0 µg/mL PI was then added to the samples, and incubate at room temperature for 30 minutes. A negative control was performed by omitting the TdT enzyme in the reaction buffer. For microscopy imaging, samples were rehydrated with PBS-T and mount with Fluoromount-G (eBioscience).

Images were collected using a Leica TCS SP8 X confocal spectral microscope imaging system with white light laser and were processed using Leica application suite advanced fluorescence lite software.

The raw number of apoptotic cells were counted manually. To avoid variation due to size difference, we defined the area of interest for each regenerating time period. In addition, the total number of a cells in a regenerating area was counted using ImageJ software. The difference in the cell counts (stained by PI) in a designated regenerating area between individuals was less than 10%.

### Gene cloning and sequence analysis

Partial sequence of *Avi-caspase X*, *Avi-caspase Y*, *Avi-Bax*, and *Avi-Bcl-xL* were identified in the NGS database (unpublished) of *A. viride*. Rapid amplification of cDNA ends (RACE) were performed as described in previous study^1^. Similarity analysis was performed by BLASTx or BLASTp. Multiple sequence alignment and phylogenetic analysis of caspase, Bax, Bcl-xL, Bak and Bok were conducted using the entire open reading frame. Multiple sequence alignemt was performed using the MUSCLE program with default parameters in MEGA 7.0. Caspase sequence used were *H. sapiens* caspase-1 isoform X1 (XP_016873882.1), *H. sapiens* Caspase 2 (AAH02427.2), *H. sapiens* Caspase 3 (CAC88866.1), *H. sapiens* caspase-4 isoform X1 (NP_001216.1), *H. sapiens* caspase-5 isoform X1 (XP_011541323.1), *H. sapiens* Caspase 6 (NP_001217.2), *H. sapiens* Caspase 7 (AAH15799.1), *H. sapiens* caspase-7 isoform X2 (XP_006718080.1), *H. sapiens* Caspase 8 (NP_001219.2), *H. sapiens* caspase-9 isoform beta (NP_001264983.1), *H. sapiens* Caspase 10 (NP_116759.2), *H. sapiens* Caspase 14 precursor (NP_036246.1), *H. sapiens* caspase-14 isoform X1 (XP_011526163.1), *X. laevis* Caspase 1b (AAI69961.1), *X. laevis* Caspase 2 (NP_001081404.1), *X. laevis* caspase-3 (NP_001081225.1), *X. laevis* Caspase 6 (NP_001081406.1), *X. laevis* caspase-7(NP_001081408.1), *X. laevis* caspase 8 L homeolog (NP_001079034.1), *X. laevis* caspase 9 S homeolog (NP_001079035), *X. laevis* Caspase 10 (NP_001081410.1), *D. melanogaster* death caspase-1 (NP_476974.1), *D. melanogaster* drICE protein (CAA72937.1), *D. japonica* caspase-2 (QDF63027.1), *D. japonica* caspase-3A (QDF63028.1), *D. japonica* caspase-6 (QDF63031.1), *D. japonica* caspase-8 (BAP74679.1), *N. vectensis* caspase-3 (XP_001633895.1), *N. vectensis* caspase-7-like isoform X2 (XP_032232761.1), and *N. vectensis* caspase-8 (XP_001626560.2).

Bcl-2 protein family sequence used were *M. musculus* Bak protein (CAA73684.1), *H. sapiens* Bak protein (AAX36890.1), *Crassostrea hongkongensis* Bcl-2-like antagonist_killer (AJO67801.1), *X. laevis* BCL2 antagonist_killer 1 (NP_001089587.1), *H. vulgaris* Bak (NP_001296708.1), *Botryllus schlosseri* Bax (AMY56534.1), *H. vulgaris* Apoptosis regulator Bax (XP_002158069.3), *M. musculus* apoptosis regulator BAX (NP_031553.1), *M. galloprovincialis* Bcl-2-associated (AGK88247.1), *H. sapiens* Bcl-xL (CAA80661.1), *M. musculus* Bcl-xL (NP_001276645.1), *U. unicinctus* Bcl-xL protein (AMT84566.1), *H. vulgaris* Bcl-2-related ovarian killer (XP_012566912.1), *X. laevis* BCL2 family apoptosis regulator (NP_001139563.1), *H. sapiens* bcl-2-related ovarian killer (NP_001253851.1), *D. melanogaster* BOK (AAF25955.1), and *D. melanogaster* death executioner Bcl-2 (AAF26841.1). We consrtucted the phylogenetic tree using the maximum likelihood method in MEGA 7.0. Tree node reliability was assessed in MEGA 7.0 using 500 bootstrap replicates.

### RNA extraction for quantitative real-time RT-PCR (qRT-PCR)

The total RNAs were extracted from 20 worms from each regenerating time pointsusing TRIzol and reverse-transcribed to cDNA using SuperScript III Kit (Invitrogen). Transcriptional levels were determined by Bio-Rad iCycler using SYBR green system (Bio-Rad). Relative quantification of gene expression was calculated by the ΔΔCt method. Three technical replicates were performed in each real-time PCR reaction, and a no-template blank was served as negative control. *Avi-Actin* is used to normalized control.

The primers were: *Avi-actin* qPCR primers (forward: 5′-ATGGAGAAGATCTGGCATCA-3′, reverse: 5′-GGAGTACTTGCGCTCAGGTG-3′); *Avi-caspase X*qPCR primers set#1 (forward: 5′-GTGCATACGCTGAATTCTCAAAGTAG-3′, reverse: 5′-CAGCTG TTACGTGAAGTACAAGAGCA-3′); *Avi-caspase X* qPCR primers set#2 (forward: 5′-CTAGCCGCAATGAGAGAGATATG-3′, reverse: 5′-GTCATTTCACACAGGCCACTTTC-3′); *Avi-caspase Y* qPCR primers (forward: 5′-GATGGAACTGACGCAGTAGATG-3′, reverse: 5′-ACGCGGTGTTGTGGAATAA-3′)

*Avi-Bax* qPCR primers (forward: 5′-GGCTGGATAATCAATCGTGGTGG-3′, reverse: 5′-CAGCCAATGTAACTCCTGTCAAG-3′); *Avi-Bcl-xL* qPCR primers (forward: 5′-CTGGGATGCCTTTGACGAATACTT-3′, reverse: 5′-TTAGATCTGCGTCTGTCCAAACAC-3′); *Avi-wnt-4* qPCR primers (forward: 5′-CTACTTTACTTACCTACGCTTCACTC-3′, reverse: 5′-CCTGACATTCATCAATGGCTACCTGT-3′); *Avi-GAPDH* qPCR primers (forward: 5′-CATGCATTTACGGCCACCCAGAAA-3′, reverse: 5′-TACACGGAACGCCATACCAGTCAA-3′); *Avi-tert* qPCR primers (forward: 5′-GCAAGTAGCCAGCGAAAGAG-3′, reverse: 5′-GCACCCACACCTCCATTATTAA-3′);

### Whole mount *in situ* hybridization (WMISH)

To prepare RNA probe for WMISH, the target sequence was amplified by PCR using specific primers. *Avi-caspase X* WMISH primer (forward: 5′-GCGTGATCTGATGGAGAAGCTACAGTT-3′, reverse: 5′-ACTCTATTTTGCATGACTGGCCG-3′); *Avi-caspase Y* WMISH primers (forward: 5′-CCACAAAAAGAGAGGCTTGTTTCTCC-3′, reverse: 5′-GATCTCCATGAGTTAGAAATGCGCAG-3′). Desired PCR products were then inserted into yT&A vector (Yeastern Biotech). Target sequence in yT&A vector was transformed into *E. coli*, and plasmid DNA was used to performed secondary PCR. *In vitro* transcription was then proceed using T7 polymerase (Ambion) and DIG-labeled rNTP (Roche). Finally, the RNA probe was 5 times diluted in HYB^+^ buffer (50% formamide, 5X saline sodium citrate (SSC), 9.2 mM citric acid, 50 µg/ml heparin, 0.5 mg/mL yeast tRNA (Sigma-Aldrich) and 0.1% Tween-20 in DEPC-H_2_O).

Samples were collected 24 hours prior to the experiment and fixed in 4% PFA at 4 °C. Samples were washed with phosphate buffered saline with 0.1% Tween-20 (PBS-T, pH 7.4) for 5 times. After wash, sample were treated with 10 mg/ml proteinase K in PBS-T buffer for 10 minutes. The proteinase K digested samples were re-fixed in 4% PFA for 20 minutes on shaker, then washed with PBS-T 5 times to remove residual PFA. Samples were then pre-hybridized in HYB^−^ buffer (HYB^+^ without yeast tRNA and heparin) for 30 minutes at 65 °C, then pre-hybridized in HYB^+^ buffer for 2 to 3 hours at 65 °C. Finally, HYB^+^ contained RNA probe was added to the samples, and the sample were hybridized at 65 °C overnight. After overnight hybridization, samples were briefly washed with HYB^−^ buffer at 65 °C to remove excessive RNA probe, and then gradually changed to 2X SSC-TW (SSC with 0.1% Tween-20) at 65 °C. Sample were then transferred into 0.2X SSC-TW for 15 minutes at 65 °C twice. After a final wash in the PBS-T, blocking buffer (5% bovine serum albumin (BSA, Sigma-Aldrich) in PBS-T) was added for 4 hours at room temperature. After blocking, the sample was transferred into antibody solution, which contained anti-DIG antibody conjugated with AP (Roche) 1:10000 diluted in blocking buffer. Samples were then incubated at 4 °C overnight. Prior to staining, samples were washed 10 times with PBS-T, 5 minutes each. Samples were transferred into staining buffer (100 nM Tris-HCl pH9.5, 50 mM MgCl_2_, 100 mM NaCl and 0.1% Tween-20 in DEPC-H_2_O). Before staining, sample were transferred to a 96 well plate containing staining solution (solution buffer contain NBT and BCIP). The sample were kept away from light during the staining process. After staining, the staining solution were washed away with PBS-T and dehydrated with methanol. For microscopy imaging, samples were rehydrated with PBS-T and mounted with Fluoromount-G. Images were collected using an Olympus DP80 microscope and were processed using Cell Sens Dimension software.

### RNA interference (RNAi)

The RNAi protocol was modified as described previously^2^. Double stranded RNA (dsRNA) was synthesized according to E-RNAi - a web application used to design optimized RNAi construct^64^. Partial sequence about 300 base pairs (bp) of *ampicillin resistant gene* (*Amp*, as MOCK group) or *Avi-caspase X* were constructed with L4440 vector (provided by Dr. Wu’s lab, Institute of Molecular and Cellular Biology, National Taiwan University, Taiwan). Then *in vitro* transcribed with T7 polymerase to produce dsRNA. Microinjecting method was modified based on previous study^65^. Borosilicate glass capillaries (World Precision Instruments) was inserted into the Flaming / Brown micropipette puller model P-97 (Sutter Instrument) to produce microinjecting pipette. The following pulling method was designed, pressure: 50, heat: 640, pull:125. The microinjector used for dsRNA delivery was Nanoliter 2000 injector (World Precision Instruments). Each injection was set at slow mode with volume of 27.6 nL. To avoid fracturing of microinjecting pipette, worms were injected on culture dish containing 1.5% agar gel. The injection was made at the 4^th^ anterior segment, in the body cavity. Successful injection will be followed by inflation of the entire body cavity. Each individual animal was injected 100 ng of dsRNA through Nanoliter 2000 injector for 2 consecutive days, then immersed in ASW containing 4 ng/µL of *Avi-caspase X *dsRNA. The solution was replaced each day to ensure its effectivity. Worms were then collected for RNA extraction or regeneration study.

### 5-ethynyl-2’-deoxyuridine (EdU) labeling for cell proliferation

Cell proliferation was monitored by *in vivo* labeling with EdU. Worms were exposed to 0.1 mM of EdU (Invitrogen) in ASW for 12 hours. For the cell proliferation experiment, worms were amputated, and incubated in EdU solution for 12 hours prior to collection. EdU incorporated during S-phase of mitosis was detected by immunohistochemistry using the Click-it EdU Alexa Fluor 488 Imaging Kit (Invitrogen) according to the manufacturer’s instructions. Specimens were mounted in Fluoromount-G and images were taken on an Olympus DP80 microscope.

### Statistical analysis

Data were test for significance using Mann Whitney U test. Probability values of P ≦ 0.05 were regarded as statistically significant.

## Supplementary information

Supplementary information.
