# Supplementary material for: Caspase dependent apoptosis is required for anterior regeneration in Aeolosoma viride and its related gene expressions are regulated by the Wnt signaling pathway
Source: Sci Rep. 2020 Jul 1;10:10692. doi: 10.1038/s41598-020-64008-1 (PMC7329817; doi:10.1038/s41598-020-64008-1)
Supplement: Supplementary file 1 — Supplementary information. [file 41598_2020_64008_MOESM1_ESM.pdf]

Supplementary information

Caspase dependent apoptosis is required for anterior regeneration in *Aeolosoma viride* and its related gene expressions are regulated by the Wnt signaling pathway

Sheridan Ke-wing Fok[1], Chiao-Ping Chen[1], Tzu-Lun Tseng[1], Yi-Hua Chiang[1], Jiun-Hong Chen[1]

[1] National Taiwan University, Department of Life Science, Taipei city 10672, Taiwan

Correspondence and requests for materials should be addressed to Jiun-Hong Chen (email: chenjh@ntu.edu.tw) or Sheridan Ke-wing Fok (email: d07b21005@ntu.edu.tw)

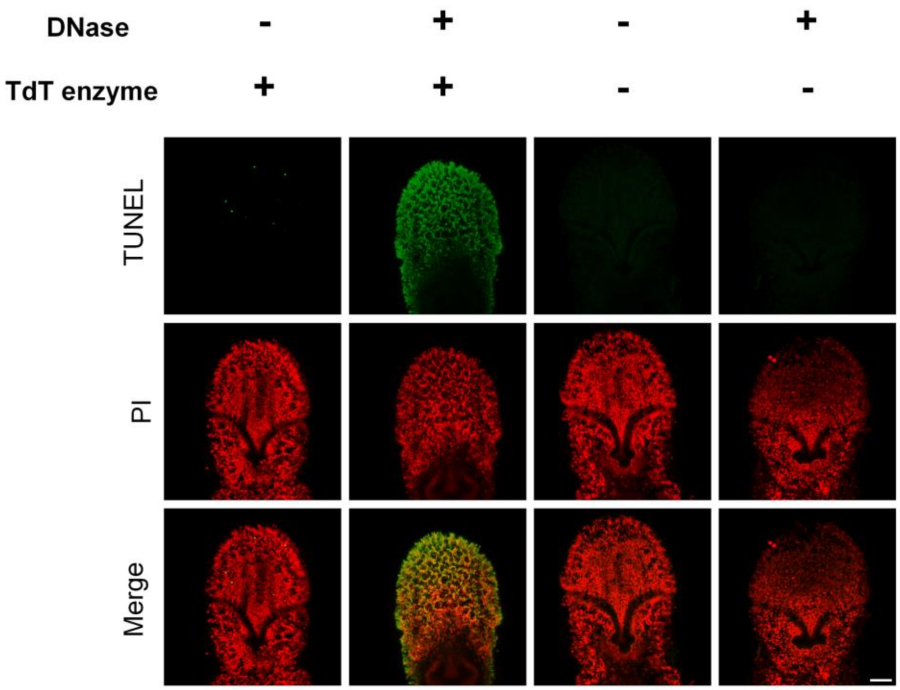

Supplementary Figure S1. Positive and negative controls of TUNEL labeling. Positive control was performed by pre-incubation with DNase I. Negative control was performed by omitting the TdT enzyme (n=10/group). Scale bar: 50 μm.

a

```
1 MENLYADEIR NPPELTGPET YHACILYSSE LINAALEIES KLIYNKFSVH TLNSQSSDDD

61 IKLYIDQSLC IIIVISGLKD ESNNNVFKIA NKKMRARLRD TAALVLHVTA VCNNFKFKLQ

121 NYKFLSNDET AIGKIDSFIL EQYKNTLNVK LPVGDVANGL AFSLFYGFLK IITPGMKNRL

181 RNNGLWKTSR NERDMALRLF IIISETCFCP PKLSDWDSAI TDGVAWTQIS ATRAGSSKRD

241 YKSTIHKLDK NSRTLYFACE LAAPIITLYD MMESGLCEMT HTEKREQMSQ FYFVLSNLIK

301 SDDNCKECVE LVYYNDAKPM KPLSDVLYDR IMKAINQDLE DDEKDSTAMK LPTGVPRENK

361 TFILTAPSHA PGYFANNVIG KYNIRAKNRG IALIISNEYF QCSQPRHGAQ ADLRKLRDLM
                                 P20 subunit
421 EKLQFKVQVE ENLTSQQICQ TLEKISKSDH SNYDCFLCFI LSHGQCGTVY GVDDKPGSEQ
                                                  SHG motif
481 VKIAQILQYF YSEFCPSLKG KPKCFFLPHC QVFDIDPNIQ PREQLPLSLQ VTDSRICGTD

541 ASNVNMSEMG NEIDNANDHM THRMNYMTIQ PGRGPVSEHV PCDEDFLVSY STLPGNFAYR

601 DPEEGTFYIQ AFVETMQRYA SMLHMIDILQ KVTEEVKRKI AALNEAERRE DALNQLPFYV
```

b

```
1 MNHKKRGLFL LFNQVNFDPS TRMGTRLGTN EDAANLMSLF QGMGFDVKRY TDRRKKDILK
           P20 subunit
61 ILQEASSYNH SDSDCFACAF LTHGDQINNE DIVFANDAYI YNRDIFAFFK GDKCSSLAGK

121 PKFFIFQACR GSNLDDGTDA VDGLDCADGD VKRRRIPIEA DFLFAYSTTP RYYSWRNQEL
         QACXG motif
181 GSWFIQDLVK VFEQHHNTLD VLSMLTLVNG KVAFEHESNS SGKMHMKRQM PCIVSQLTKH

241 FYLKNKTI
```

Supplementary Figure S2. Sequence analysis of *Avi-caspase X* and *Avi-caspase Y*.

Conceptual amino acid sequence deduced from the cDNA sequence encoding *Avi*-caspase X (a) and *Avi*-caspase Y (b). Yellow underlined region indicated the P20 subunit.
